# Screening Bambara Groundnut (*Vigna subterranea* L. Verdc) Genotypes for Drought Tolerance at the Germination Stage under Simulated Drought Conditions

**DOI:** 10.3390/plants11243562

**Published:** 2022-12-16

**Authors:** Sithembile Kunene, Alfred Oduor Odindo, Abe Shegro Gerrano, Takudzwa Mandizvo

**Affiliations:** 1 School of Agricultural, Earth and Environmental Sciences, University of KwaZulu-Natal, Private Bag X01, Scottsville, Pietermaritzburg 3209, South Africa; odindoa@ukzn.ac.za (A.O.O.); takudzwamandizvo@gmail.com (T.M.); 2 Agricultural Research Council, Vegetables, Industrial and Medicinal Plants, Private Bag X293, Pretoria 0001, South Africa; agerrano@arc.agric.za; 3 Department of Plant Sciences and Plant Pathology, Montana State University, Bozeman, MT 59717, USA; 4 Food Security and Safety Focus Area, Faculty of Natural and Agricultural Sciences, North-West University, Private Bag X2046, Mmabatho 2735, South Africa

**Keywords:** polyethylene glycol (PEG), seedling establishment, legume, smallholder farmers

## Abstract

Bambara groundnut (*Vigna subterranea* L. Verdc) is grown by smallholders and subsistence farmers in the marginal parts of sub-Saharan Africa. This legume is native to Africa and is cultivated throughout semi-arid sub-Saharan Africa. It is hardy and has been recognized as a nutritious food source in times of scarcity. Drought can negatively affect the germination or establishment of seedlings in the early stages of crop growth. Drought can limit the growing season of certain crops and create conditions that encourage the invasion of insects and diseases. Drought can also lead to a lack of crop yield, leading to rising food prices, shortages, and possibly malnutrition in vulnerable populations. A drought-tolerant genotype can be identified at the germination stage of Bambara groundnut by screening for drought-tolerance traits, and this knowledge can be applied to Bambara crop improvement programs to identify drought-tolerant traits during early growth phases. As an osmolyte, polyethylene glycol (PEG 6000) reduced water potential and simulated drought stress in Bambara groundnut seeds of different genotypes. Osmolytes are low-molecular-weight organic compounds that influence biological fluid properties. In this study, 24 Bambara groundnut genotypes were used. Data were collected on seed germination percentage (G%), germination velocity index (GVI), mean germination time (MGT), root dry mass (RDM), root fresh mass (RFM), and seven drought tolerance indices: mean productivity (MP), tolerance index (TOL), geometric mean productivity (GMP), stress susceptibility index (SSI), yield index (YI), yield stability index (YSI), stress tolerance index (STI) as well as seed coat color measurements. The data were applied to the mean observation of genotypes under simulated drought conditions (Ys) and the mean observation of genotypes under controlled conditions (Yp). Germination%, germination velocity index (GVI), mass germination time (MGT), and root fresh mass (RFM) differed significantly (*p* < 0.001) between the two stress conditions. Bambara genotypes Acc 82 and Acc 96 were found to be the most drought-tolerant.

## 1. Introduction

Bambara groundnut (*Vigna subterranea* L. Verdc) is an important legume that contributes to food and nutritional security [1]. The crop is rich in proteins and carbohydrates [2]. It is likely that Arabs introduced the Bambara groundnut to Madagascar very early [3]. Early in the seventeenth century, it reached Brazil and Surinam and then moved to the Philippines and Indonesia [3]. Arid savannah zones have seen renewed interest in the cultivation of Bambara groundnut in recent years [4]. A semi-arid region of sub-Saharan Africa has been cultivating Bambara groundnut for centuries. Abiotic factors such as drought can affect Bambara groundnut production. Owing to the scarcity of water and the effects of climate change in sub-Saharan Africa, research has focused on strategies to improve the productivity of Bambara groundnuts under water stress [5]. According to a study by Khan, Rafii [6], limited research has been conducted to improve Bambara groundnuts. Researchers have conducted only scattered studies in South Africa [7]. Bambara groundnut in South Africa has not been sufficiently studied for its drought tolerance, physiology, and agronomy. Muhammad, Rafii [8] highlighted that food and nutritional security could be addressed by developing underutilized and future crops, such as Bambara groundnut, with abundant genetic resources and potentially advantageous traits.

Germination may be low when the seeds are of poor quality [9]. Several biochemical and molecular processes are involved in plant responses to drought stress [10]. As a result of drought stress regimes, physiological processes such as cellular respiration, photosynthesis rate, mineral nutrition, enzyme activity as well as redox (oxidation/reduction) homeostasis are affected. Under water-limited conditions, biochemicals, such as membrane lipoproteins, DNA, and cellular proteins, are degraded [11]. A plant’s ability to survive drought stress is based on a variety of biochemical, structural, and molecular mechanisms, including the accumulation of osmolytes like proline, proteins, sugars, and glycine betaine. High germination rates can be achieved using high-quality seeds. Germination and production are slow with poor-quality seeds, which results in poor plant stands. Seed scarring and coat color also affect the quality of Bambara groundnut seeds [12]. As seeds germinate, it is important to consider seed coat color and texture. The seed coat creates barriers that inhibit oxygen diffusion and water uptake during the first stage of germination (imbibition) [13]. As carbon dioxide diffuses from the stomata into leaves and ultimately into the cells, oxygen diffusion plays an important role in photosynthesis [14]. A major benefit of the Bambara crop is its popularity with smallholders in subsistence agriculture and smallholder farming systems, but poor seed quality will delay and affect the emergence of seedlings, which will reduce yields and plant population [15]. For low- and middle-income countries who are unable to access animal proteins, Bambara groundnut is a valuable resource for carbohydrates, essential amino acids, proteins, and energy [16]. In dry form, it consists of 51–70% carbohydrate, 6–12% moisture, 18.0–24.0% protein (high in lysine and methionine), 5.0–7.0% fat, and 5.0–12.0% protein [17]. The plant is also useful in maintaining the plant habitat because it increases soil fertility and increases the yields of other crops around it without requiring fertilizer. Therefore, it is an alternative source of food and income in the face of climate change’s negative effects [18]. Plant cells and the canopy are affected by drought in several ways. Under water stress, a crop’s growth rate may be severely reduced, resulting in a small leaf area and reduced total dry matter [19]. A better understanding of drought tolerance at seedling stage may reduce the risk of poor stand establishment during droughts and salt stress and allow for more uniform growth under conditions of irregular rainfall and drought [20].

Germination tests on Bambara groundnuts have shown that drought tolerance traits are detectable [21]. Drought can affect yield performance during seed germination and seedling establishment; thus, the ability to identify them is crucial [22]. Bambara groundnut genotypes can therefore be tested for drought tolerance during germination and seedling establishment using polyethylene glycol (PEG) [23]. Seeds grow less well in water when exposed to polyethylene glycol (PEG) [23]. During the germination phase, seed water potential drops considerably [24].

The seeds cannot absorb water because the surrounding solution has a lower water potential owing to the addition of PEG. Plant breeders can select drought-tolerant traits at early stages of development and growth and discover drought-tolerant crops for high productivity by identifying drought-tolerant traits at this stage of growth and development. Hence, the objective of this study was conducted using polyethylene glycol (PEG 6000) to evaluate Bambara groundnut genotypes for their drought tolerance.

## 2. Results

Among the genotypes, there was significant variation in germination percentage (G%), germination velocity index (GVI), the number of seeds germinated (NSG), and mean germination time (MGT). There was also significant variation in germination percentage (G%), germination velocity index (GVI), and mean germination time (MGT) with respect to the number of days. There were significant differences between the treatments in terms of germination percentage (G%), germination velocity index (GVI), mean germination time (MGT), and the number of seeds germinated (NSG). Furthermore, in terms of genotype-treatment interactions, germination percentage (G%), germination velocity index (GVI), mean germination time (MGT), and the number of germinated seeds (NSG) varied significantly, as shown in Table 1.

### 2.1. Seed Coat Color

Seed coat redness, greenness, saturation, and lightness were significantly different (*p* < 0.001) among the genotypes. Redness was the highest in Acc 95 (160.67), followed by Acc 61 (145.67) (Table 2). The genotypes used ranged in redness from 19.00 to 160.67, with Acc 200 having the lowest redness. The greenness of the genotypes used ranged from 13.00 (Acc 179) to 165.33 (Acc 100), while the blueness ranged from 6 (Acc 105) to 48.00 (Acc 25). In terms of hue, there were significant differences (*p* < 0.05) between genotypes. The hue varied between 15.33° (Acc 97) and 242.67° (Acc 200) hues. A saturation range of 23% (Acc 200) to 93.33% (Acc 105) was observed among the genotypes. The highest lightness was obtained for Acc 100 (75.5%). This was followed by Acc 61 (57.33%) and Acc 25 (57.00%). The RAL of the genotypes ranged from 5011 to 1011, with Acc 179 having the lowest RAL and Acc 95 having the highest RAL, as shown in Table 2 below.

### 2.2. Seed Germination

**Figure 1:**
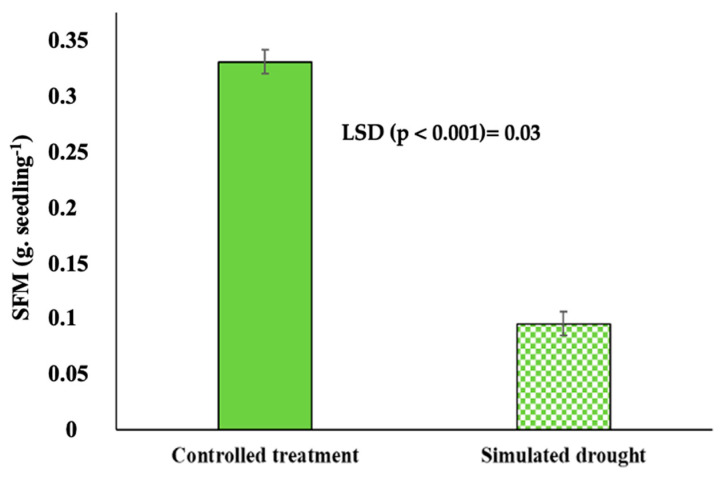
Seedling fresh mass (SFM) of seeds subjected to simulated drought conditions using 5% PEG compared to a control treatment using distilled water.

Compared to both the control treatment and the simulated drought treatment, genotypes showed highly significant (*p* < 0.001) differences in germination percentage (Table 3). Bambara groundnut seeds germinated using 5% PEG to simulate drought conditions and showed reduced germination and seedling fresh mass. Furthermore, seedling fresh mass (SFM) differed significantly (*p* < 0.001) between the two conditions (Figure 1).

### 2.3. Effect of Simulated Drought Stress on Seedling Vigor

**Figure 2:**
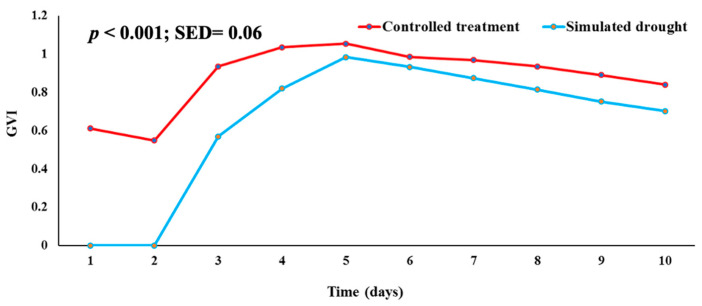
Germination velocity index (GVI) in each day subjected to simulated drought conditions using 5% PEG compared to a control treatment using distilled water.

**Figure 3:**
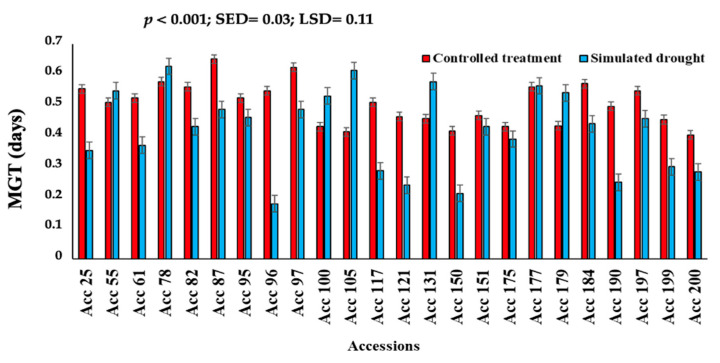
Mean germination time (MGT) of twenty-four Bambara groundnut genotypes subjected to simulated drought conditions using 5% PEG compared to a control treatment using distilled water.

In terms of germination velocity index (GVI), genotypes were significantly different (*p* < 0.001) with respect to the number of days from the beginning of the experiment (Day 0) (Figure 2). Under the controlled treatment, GVI ranged from 0.693 to 1.327, whereas simulated drought GVI ranged from 0.245 to 1.019. In most genotypes, the germination velocity index was reduced under drought conditions, except for Acc 55, Acc 131, Acc 100, and Acc 179. These genotypes can be considered drought-tolerant. Mean germination time (MGT) also differed significantly (*p* < 0.001) among the genotypes (Figure 3). A range of 0.40 to 0.65 was observed for mean germination time under controlled treatment conditions and from 0.21 to 0.63 under simulated drought conditions.

### 2.4. Drought Indices

Seven drought tolerance indices (Table 4) were calculated based on the Bambara seedling observation of each genotype under controlled treatment (Yp) and simulated drought conditions (Ys) to identify drought-tolerant and drought-sensitive genotypes. There was a higher tolerance (TOL) value of ≥ 0.354 for Acc 82 (0.354) and Acc 96 (0.348), and lower TOL values of ≤ 0.200 for Acc 61 (0.189), Acc 95 (0.198), Acc 97 (0.124), Acc 100 (0.123), Acc 105 (0.143), Acc 175 (0.162), and Acc 184 (0.059). Stress tolerance index (STI) values ≥ 0.0402 were recorded in Acc 82, Acc 151, and Acc 179, whereas STI values ≤ 0.113 were recorded in Acc 96, Acc 121, Acc 150, Acc 190, and Acc 200. Acc 78, Acc 131, Acc 151, and Acc 179 recorded geometric mean productivity (GMP) ≥ 0.192, whereas GMP values ≤ 0.111 were recorded for Acc 121, Acc 150, Acc 190, and Acc 199. Higher YI values of ≥ 1.317 were recorded for Acc 61 and Acc 55, whereas lower YI values of ≤ 0.471 were recorded for Acc 96, Acc 121, Acc 150, and Acc 190.

### 2.5. Principal Component Analysis for Assessed Traits under Simulated Drought and Controlled Conditions

For the evaluated traits, Table 5 shows the PCA with factor loadings, eigenvalues, and percentage variances. Under controlled treatment conditions, PC 1 accounted for 28.36% of the total variation and was positively correlated with MPI, STI, GMP, YI, YSI, RDM, RFM, GVI, MGT, Red, Green, Blue, Saturation (%), lightness (%), and germination (%). PC 2 positively correlated with RAL, Hue, and TOL, contributing 23.27% of the total variation. PC 3 accounted for 18.87% of the total variation and was positively correlated with STI, GMP, YI, YSI, GVI, MGT, Hue, RAL, and germination%. TOL, GVI, MGT, Red, saturation %, lightness, RAL, and germination % were positively correlated with PC 4, which accounted for 13.25% of the total variation. Germination %, TOL, RFM, GVI, MGT, Red, Green, and Blue were positively correlated with PC 5, accounting for 8.56% of the total variation.

Under simulated drought conditions, PC 1 accounted for 32.39% of the total variation and was positively correlated with MPI, STI, GMP, YI, YSI, RFM, GVI, MGT, Red, Green, Blue, and germination. Red, Green, Blue, Saturation (%), and lightness (%) were positively correlated with PC 2, accounting for 21.91% of the total variation. PC 3 accounted for 17.89% of the total variation and positively correlated with TOL, MPI, STI, GMP, RDM, RFM, GVI, and Red.

Green, saturation (%), lightness (%), and RAL. YI, YSI, RDM, GVI, MGT, Red, Saturation (%), lightness (%), RAL, and Germination (%) were positively correlated with PC 4, which accounted for 10.49% of the total variation. RDM, GVI, MGT, Red, Green, Blue, Hue (°), lightness (%), and RAL were positively correlated with PC 5, which accounted for 5.77% of the total variation. 

**Figure 4:**
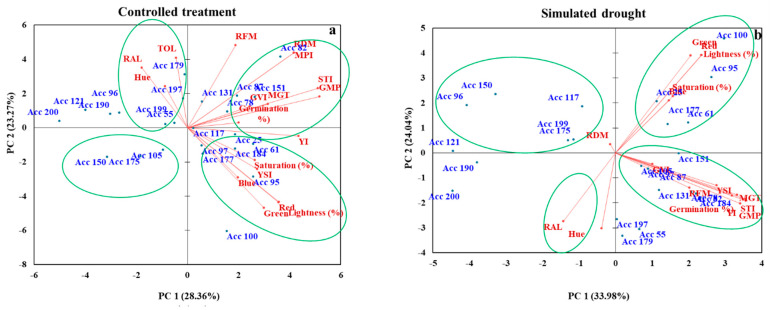
Biplot of drought indices, seed coat color, seed growth, and seed vigor based on the first two principal components axes (PC 1 and PC 2) for 24 Bambara genotypes in controlled treatment (**a**) and simulated drought conditions (**b**). SSI: stress susceptibility index, TOL: stress tolerance, MP: mean productivity, STI: stress tolerance index, GMP: geometric mean productivity, YI: yield index, YSI: yield stability index, RAL, Hue, Red: redness, Blue: blueness, Green: greenness., MGT: mean germination time, GVI: germination velocity index, RDM: roots dry mass, RFM, roots fresh mass.

PC biplots based on PCA analysis were used to determine the relationship between Bambara groundnut genotypes based on the evaluated physiological and morphological parameters under CT (Figure 4a) and SD conditions (Figure 4b). Traits represented by parallel vectors or those close to each other revealed a strong positive association. Those located nearly opposite (at 180°) showed a highly negative association, and the vectors toward the sides showed a weak relationship. Under CT conditions, genotypes Acc 55, Acc 179, Acc 197, and Acc 199 were grouped based on high TOL, RAL, and Hue. Acc 78, Acc 82, Acc 87, and Acc 151 were grouped based on their high germination %, RDM, MPI, STI, GMP, GVI, and MGT. Acc 25, Acc 61, Acc 95, Acc 97, Acc 177, and Acc 184 were grouped based on high saturation (%), YSI, Blue, Lightness, Green, and Red. Acc 105, Acc 150, and Acc 175 were not associated with any of the traits.

Under SD conditions, Acc 78, Acc 97, Acc 82, Acc 105, Acc 131, Acc 151, and Acc 194 were grouped based on their high GVI, RFM, YSI, MGT, STI, GMP, and germination%. Acc 25, Acc 61, Acc 95, and Acc 100 were grouped based on high Blue, Saturation%, Lightness, Red, and Greenness. Acc 96, 117, 150, 175, and 199 were not associated with any of the traits evaluated in the second quadrant. By contrast, RAL and Hue were not associated with any of the genotypes in the fourth quadrant.

### 2.6. Pearson Correlations of Trait Assessments under Simulated and Controlled Drought Conditions

**Figure 5:**
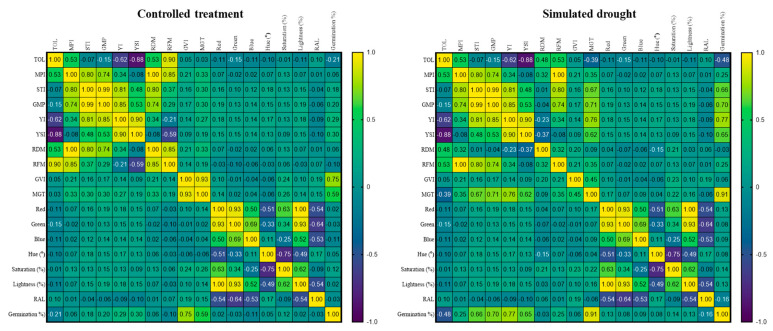
Correlations of drought indices, seed coat color, seed growth, and seed vigor based on the first two principal component axes (PC 1 and PC 2) for the 24 Bambara genotypes under controlled treatment and simulated drought conditions. SSI: stress susceptibility index, TOL: stress tolerance, MP: mean productivity, STI: stress tolerance index, GMP: geometric mean productivity, YI: yield index, YSI: yield stability index, RAL, Hue, Red: redness, Blue: blueness, Green: greenness., MGT: mean germination time, GVI: germination velocity index, RDM: roots dry mass, RFM, roots fresh mass.

An analysis of the Pearson correlation coefficients between morphological and physiological traits exhibited by Bambara groundnut genotypes under controlled treatment (CT) and simulated drought conditions (SD) is presented in Figure 5. Under CT conditions, significant positive correlations were observed between STI and GPM (*r* = 0.99), TOL and RFM (*r* = 0.90), MPI and RDM (*r* = 1.00), MPI and RFM (*r* = 0.85), STI and YI (*r* = 0.81), STI and RDM (*r* = 0.80), GMP and MPI (*r* = 0.74), GMP and YI (*r* = 0.85), GMP and RDM (*r* = 0.74), YI and YSI (*r* = 0.90), RDM and MPI (*r* = 0.80), RDM and RFM (*r* = 0.85), RFM and TOL (*r* = 0.90), RFM and MPI (*r* = 0.85), germination% and GVI (*r* = 0.75), Lightness and Red (*r* = 1.00), Lightness and Green (*r* = 0.93), and Green and Red (0.95). There was a significant negative correlation between YSI and TOL (*r* = −0.88), as well as between Hue and Saturation % (*r* = −0.75). 

Under SD condition, significant and positive correlations were observed between MPI and STI (*r* = 0.80), MPI and GMP (*r* = 0.74), STI and GMP (*r* = 0.99), STI and YI (*r* = 0.81), MPI and RFM (*r* = 1.00), GMP and YI (*r* = 0.85), GMP and RFM (*r* = 0.74), GMP and MGT (*r* = 0.71), YSI and YI (*r* = 0.90), YI and GMP (*r* = 0.76), YI and germination % (*r* = 0.77), GMP and germination % (*r* = 0.70), RFM and MPI (*r* = 1.00), RFM and STI (*r* = 0.80), RFM and GMP (*r* = 0.74), MGT and GMT (*r* = 0.71), MGT and YI (*r* = 0.76), Red and Green (*r* = 0.93), Red and Lightness% (*r* = 1.00), MGT and Germination % (*r* = 0.91), Green and Lightness %(0.93). Significant and negative correlations were observed between TOL and YSI (*r* = −0.88), Hue and Saturation% (*r* = −0.75)

## 3. Discussion

This study investigated whether polyethylene glycol (PEG 6000) can be used to simulate drought conditions to screen Bambara groundnut genotypes for drought tolerance at the early growth stages during germination and seedling establishment. Furthermore, this study attempted to identify traits associated with drought tolerance and seed coat color in Bambara groundnuts. In terms of seed coat color, there was a highly significant difference (*p* < 0.001) between genotypes in terms of redness, greenness, saturation, and lightness. These results are in agreement with those obtained by Mandizvo and Odindo [12]. Their study revealed that seed coat thickness and color alone could not explain seed hydration patterns. They highlighted that darker and lighter-colored genotypes absorbed water slowly. Compared to seeds grown in distilled water, those exposed to simulated drought conditions showed significantly lower germination percentages and seed vigor. With respect to root fresh mass, the highly significant difference between the control treatment and simulated drought conditions could be attributed to PEG addition reducing water potential [25], which affected dry matter accumulation. Additionally, the two treatments differed significantly in terms of germination percentage over the course of 10 days (*p* < 0.001) Table 3. By altering the biochemical and physiological processes during the first and second phases of germination, osmotic compounds such as polyethylene glycol (PEG) can simulate drought stress and decrease the germination percentage [26]. The germination rates of Acc 78 and Acc 131 were highest under simulated drought conditions (63% and 58%, respectively). There is no guarantee that a seed is drought-tolerant simply because it has a high germination rate [27]. Zondi [28] reported that high germination does not always equate to rapid and uniform germination or vigorous stands once germination begins. Their study revealed that it is difficult to separate the performance of Bambara landrace seeds from standard germination. For this reason, germination rate and vigor are useful indicators of separation. For seeds subjected to simulated drought conditions, germination was slow during the first three days (Table 3). Gao, Bamba [29] reported similar results and attributed the differences to seeds that were more vigorous under non-stressed conditions (control) than under simulated drought stress conditions. They highlighted that a breeding program for Bambara groundnuts makes use of yield-related traits to develop structured populations and breeding lines. Water potential was significantly reduced in the PEG solution, causing seed imbibition to be hindered, cell division to be reduced, and water uptake to be impaired, resulting in a decrease in germination [30]. Mayes, Ho [30] also reported that it is possible to use Bambara groundnut to contribute to a climate-change-resilient agricultural system. Particularly in low-input farming, nitrogen-fixing and stress-tolerant legumes are essential. It is still challenging to make greater use of this legume by addressing existing negative traits and developing markets and products. Different genotypes germinate at different rates under drought stress (lower water potential) [31]. They showed that the decrease in the osmotic potential of germination media leads to an increase in seeding proline content. The genotypes tested showed highly significant differences (*p* < 0.01) in terms of germination velocity index (GVI) (Figure 2) and mean germination time (MGT) (Figure 3). This suggests that genotypes are highly genetically variable [29]. Poor seedling establishment reduced plant populations, and decreased yield can be the result of slow and prolonged seed germination. Guo, Du [32] found that seed quality is more closely related to seed color than to seed type. They confirmed that seed viability does not necessarily imply good seed vigor. Seed germination is also affected by excessive water intake [33]. During seed germination, oxygen is required to fuel its metabolism until it transitions to an autotrophic state when photosynthesis is used [34]. Acc 82 and Acc 96 showed the highest drought-tolerant with TOL of (0.354 and 0.348) under controlled treatment conditions and simulated drought conditions (Table 4). In addition to their high tolerance, these genotypes also exhibit high seedling growth of (0.461 and 0.368 g/seedling), respectively. There was a good correlation between these genotypes and drought tolerance because they could imbibe water at lower water potentials. A range of genotypes was found to be the most sensitive, resulting from the analysis of these indices. Different genotypes show different tolerances to stress owing to their genetic and physiological abilities to influence water absorption in plants [35]. Khakwani, Dennett [35] found that the first stage of growth to be affected by water deficit was seed germination. Our results also showed that Acc 82 had the highest stress tolerance index (STI) Table 4. The mean productivity (MP) and stress tolerance index (STI) are the most reliable indices for selecting Bambara groundnut genotypes [36]. According to principal component analysis, Acc 82 had the highest PC 2 and were highly correlated with RDM and MPI (Figure 4). These genotypes also show considerable potential for improved drought tolerance. High drought susceptibility and low yield stability were associated with Acc 150, Acc 105, and Acc 150 (Figure 4). PCA is widely used to determine the diversity of phenotypic characteristics and to group them based on similar features. The lowest PC 1 and PC 2 scores were found in Acc 121, Acc 190, and Acc 200. Ahmadizadeh, Valizadeh [37] also reported similar results when investigating durum wheat genotype behavior under normal irrigation and drought stress conditions in a greenhouse in 2012. PCA analysis has shown that PCA was effective in confirming the main parameters and estimating drought tolerance levels of different genotypes at the germination stage in a wide variety of crops [23]. In their study, they found that some genotypes are more drought tolerant than others based on different drought indices. Their results were confirmed by biplots and cluster analysis. Using PCA, we estimated the drought tolerance levels of Bambara genotypes using seven drought indices: means productivity (MP), stress tolerance index (STI), geometric mean productivity (GMP), tolerance index (TOL), stress susceptibility index (SSI), and yield index (YI). From 24 genotypes, Acc 82, and Acc 96 showed highest drought tolerance (Table 5 and Figure 4). Based on these indicators, it is possible to determine whether a genotype is drought-tolerant, moderately tolerant, or drought-susceptible [38]. There is a highly significant correlation between the stress susceptibility index (SSI) and the tolerance index (TOL). As the stress susceptibility index increased, tolerance also increased. Mean productivity (MP) and stress tolerance index (STI) were highly correlated. In contrast, the stress tolerance index (STI) and yield index (YI) have highly significant correlations with each other. The analysis of the main components can be performed using correlated parameters [23]. Significantly correlated parameters can be used to determine the drought tolerance levels of different genotypes based on the significant correlations between them [39]. Researchers have used correlation to combine traits for genotype selection programs to make the best selection [40]. Kamali, Jahanbakhshi [41] emphasized that alterations in parameters and indices can be used to determine drought tolerance.

## 4. Materials and Methods

### 4.1. Plant Material

**Figure 6:**
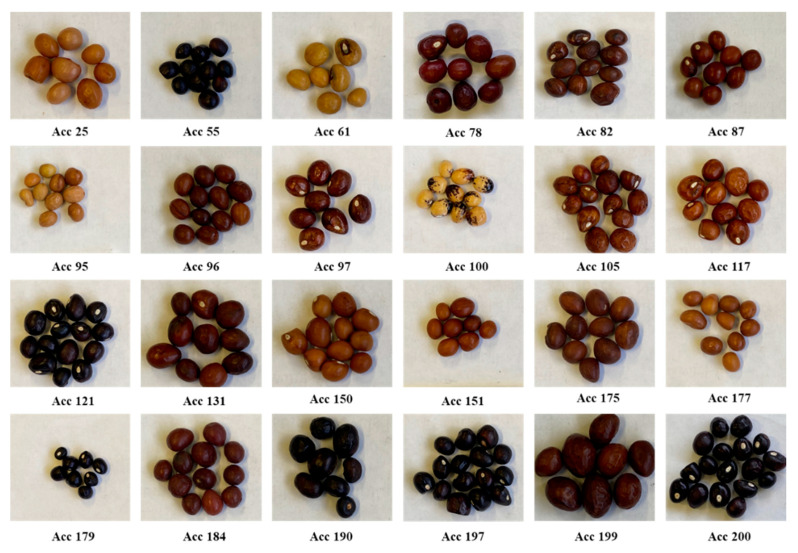

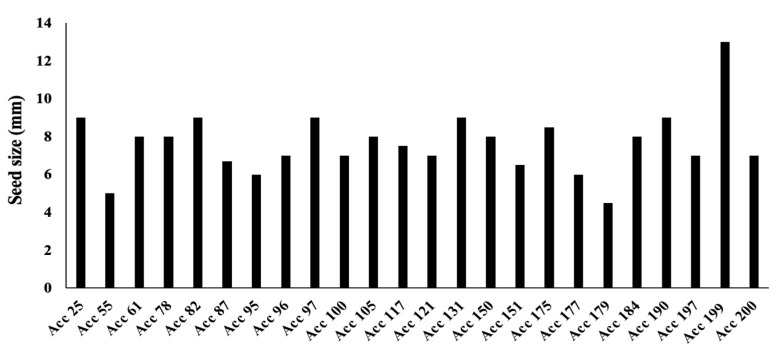
Twenty-four Bambara groundnut genotypes were used in the study with their seed sizes.

Twenty-four Bambara groundnut genotypes (Figure 6) were obtained from the Agricultural Research Council (ARC) GenBank. Genotypes were obtained from seeds harvested from previous studies. The genotypes were categorized into different seedcoat seed coat colors which are; ochre brown, graphite black, red brown, sepia brown, brown beige, mahogany brown, golden yellow, red brown, clay brown, jet black, fawn brown, signal brown, ochre brown, steel blue, terra brown, and brown olive. In terms of size, Acc 199 had the largest size, followed by Acc 25 and Acc 82. Results of drought indices (Table 6) classify Acc 82 and Acc 96 as highly drought tolerant. The seeds were assessed at the Seed Science Laboratory, School of Agricultural Earth and Environment (SAEES), University of KwaZulu-Natal, Pietermaritzburg, South Africa. 

### 4.2. Seed Coat Color

Seed coat color measurements were performed on Bambara groundnut genotypes using a stereomicroscope integrated with computer software (Leica Application Suite 4.0, South Africa). Hue, saturation, and lightness measurements were recorded and converted from red, green, and blue (RGB) codes (HSL) [12].

### 4.3. Hue, Saturation, and Lightness (HSL)

A standardization of RGB values was used to convert tri-stimulus values (RGB) to HSL, as described in (Equations (1)–(6)).

(1) $$nr=\frac{R}{R+G+B}$$

(2) $$ng=\frac{G}{R+G+B}$$

(3) $$nb=\frac{B}{R+G+B}$$

where *nr*, *ng*, and *nb* are normalized values between 0 and 1 with *nr* + *ng* + *nb* = 1. Transformation to HSL was achieved using the following equations: Standard germination test

(4) $$H=\left[2\pi \right]-\cos^{-1}\left(\frac{0.5\times \left(\left(nr-ng\right)+\left(nr-nb\right)\right)}{\sqrt{\left(\left(nr-ng\right)2\right)+\left(nr-nb\right)\left(ng-nb\right))}}\right)$$

(5) $$S=1-3\times minimum\;\left(nr,ng,nb\right)$$

(6) $$L=\frac{R+G+B}{3\times 255}$$

### 4.4. Germination

Standard germination tests were conducted for different seed coat color categories. The seeds were germinated under non-stress conditions (distilled water with no PEG solution, 0%) as well as under drought-stress conditions (5% PEG solution). The 5% PEG was chosen based on Pavlis, 2020 [42] with good results when investigating the effects of PEG on soybean germplasm at 5%. During the simulation of drought stress, PEG concentrations were determined based on Rahmah, Ilyas [23]. A 35 × 30 cm brown germination filter paper was used to grow Bambara groundnut seeds. For the control treatments, distilled water was used to moisten the germination paper, whereas a PEG solution was used for simulated drought treatments. In each filter paper, approximately 60 mL of water and peg solution were used. The seeds were washed three times with distilled water according to the procedure described by Rahmah, Ilyas [23]. Ten (10) seeds were used on each filter paper. The genotypes were replicated three times. Seeds were germinated at 25 °C temperature and 95% humidity using a Light and Seed Germination Incubator. Newly germinated seeds were counted every day when they protruded at least 2 mm from the radicle. Observations under drought-stressed and non-stressed conditions (*Ys* and *Yp*) were calculated by weighing the fresh mass of germinated seedlings for each genotype based on average observations under drought-stress conditions (*Ys*) and observations under non-stressed conditions (*Yp*). Micro cw30 digital bench scale was used to measure fresh mass on day 10 for simulated drought and control treatments. Based on the number of germinated seeds on each filter paper multiplied by 100, the percentage of germinated seeds was calculated. Germination velocity, defined by the germination velocity index (*GVI*), was calculated according to the method of [21].

(7) $$GVI=\frac{G_{1}}{N_{1}}+\frac{G_{2}}{N_{2}}+\ldots +\frac{G_{n}}{N_{n}}$$

where: *GVI* = germination velocity index; *G*_1_, *G*_2_…*G_n_* = number of germinated seeds in first, second, and last count; and *N*_1_, *N*_2_…*N_n_* = number of germinating days at the first, second, and last counts, respectively. Mean germination time was calculated as described by Koné, Koné [43].

(8) $$MGT=\frac{\sum Dn}{\sum_{n}}$$

where: *MGT* = mean germination time, *n* = the number of seeds that were germinated on day *D*, and *D* = the number of days counted from the beginning of germination.

### 4.5. Drought Indices

Seven selection indices; mean productivity (MP), stress tolerance index (STI), geometric mean productivity (GMP), tolerance index (TOL), stress susceptibility index (SSI), yield index (YI), and yield stability index (YSI) were estimated for each genotype based on observations under non-stress (*Ys*) and drought-stress (*Yp*) conditions. Quantitative drought resistance indices were calculated using the following formulas:

**Table 6:** Drought tolerance indices used to evaluate Bambara groundnut genotypes for drought tolerance.

Drought Tolerance Indices	Equation	Reference
Stress Susceptibility Index (SSI)	$\mathrm{SSI}=\frac{\left[1-\frac{Ys}{Yp}\right]}{\left[1-\frac{\bar{Y}s}{\bar{Y}p}\right]}$	[44] [Equation (4)]
Tolerance (TOL)	$\mathrm{TOL}=Y_{p}-Y_{s}$	[45] [Equation (5)]
Mean Productivity Index (MPI)	$\mathrm{MPI}=\frac{Y_{p}+Y_{s}}{2}$	[46] [Equation (6)]
Stress Tolerance Index (STI)	$\mathrm{STI}=\frac{Y_{p}\times Y_{s}}{{\left({\bar{Y}}_{p}\right)}^{2}}$	[47] [Equation (7)]
Geometric Mean Productivity (GMP)	$\mathrm{GMP}=\sqrt{\left(Y_{p}\right)\left(Y_{s}\right)}$	[48] [Equation (8)]
Yield Index (YI)	$\mathrm{YI}=\frac{Ys}{\bar{Y}s}$	[49] [Equation (9)]
Yield Stability Index	$\mathrm{YSI}=\frac{Y_{s}}{Y_{p}}$	[50] [Equation (10)]

### 4.6. Data Analysis

GenStat^®^ (VSN International, Harpenden, UK, 1968) was used to analyze the data using analysis of variance (ANOVA). Tukey’s test was used at a significance level of 5% to separate the means. Principal components were analyzed using XLSTAT (Data Analysis and Statistical Solution for Microsoft Excel, Addinsoft, Paris, France, 2017). GraphPad Prism Version 9.2.0, 2021 (GraphPad Software, Inc., San Diego, CA, USA) was used for the correlation analysis. In this study, we examined whether there were any correlations or associations between the drought indices of 24 Bambara groundnut genotypes.

## 5. Conclusions

This study confirmed that drought stress can negatively affect the seedling growth of Bambara groundnut, an underutilized indigenous crop in South Africa. Based on seed vigor, seedling fresh mass, germination and drought indices, and seed coat color, PEG may provide a screening tool to identify drought-tolerant genotypes during germination. Understanding Bambara groundnut landrace seed quality is important for increasing yield and yield-related traits under stress conditions. Through the use of polyethylene glycol (PEG) during the germination phase, this study is expected to contribute to improving Bambara groundnut production and food security through the use of PEG during the germination phase. Acc 82 and Acc 96 were identified as drought-tolerant genotypes using drought indices, PCA, biplot analysis, and correlation analysis. Drought stress and seed coat color may impact the germination of Bambara groundnuts when the seeds are at the germination and seedling establishment stages. In order to be able to speak of gains in productivity after the seedling period, similar experiments will have to be carried out in phenological stages subsequent to the seedling period. The germination stage and seedling establishment are crucial for Bambara production. Germination papers containing 5% PEG 6000 solution can be used to evaluate Bambara groundnut seed germination.

## Figures and Tables

**Table 1:** An analysis of 24 Bambara groundnut genotypes under controlled and simulated drought conditions. The mean square of the germination percentage (G%), the germination velocity index (GVI), the mean germination time (MGT), seedling fresh mass (SMF), and the number of seeds germinated (NSG).

Source of Variation	d.f	G (%)	GVI	MGT	SFM	NSG
Rep	2	10662.7	10.1981	1.06627	0.118	106.627
Genotype	23	3801.9 **	1.3405 **	0.38019 **	0.00583	38.019 **
No. of days	9	110804.4 **	9.8958 **	11.08044 **		1108.044 **
Treatment	1	26608.4 **	19.9866 **	2.66084 **	1.73356	266.084 **
No. of days. Genotypes	207	209.2	0.1446	0.02092		1.368
Genotypes. Treatment	23	2832.2 **	1.2271 **	0.28322 **	0.00838	28.322 **
Genotypes. No. of days. Treatment	207	1	1	1		1
Residual	958	300.8	0.2506	0.03008	0.00908	3.008
Total	1439					

G(%); germination percentage, GVI; germination velocity index, MGT; mean germination time, SFM; seedling fresh mass, NSG; number of seeds germinated, DF; degree of freedom, Rep; replication, ** Significant at the 0.01 probability level, MS; mean square.

**Table 2:** Seed coat color among twenty-four Bambara groundnut genotypes.

Genotypes	Red	Green	Blue	Hue (^o^)	Saturation (%)	Lightness (%)	RAL	Name of Color
Acc 25	144.67	98.67	48.00	31.33	67.33	57.00	8001	Ochre brown
Acc 55	25.67	21.33	16.33	95.00	47.33	10.33	9011	Graphite black
Acc 61	145.67	102.33	13.00	40.67	91.00	57.33	8001	Ochre brown
Acc 78	87.67	34.33	19.33	129.67	80.00	34.33	8012	Red brown
Acc 82	84.33	49.00	35.67	131.00	63.00	33.00	8014	Sepia brown
Acc 87	84.33	37.33	8.00	23.00	90.67	33.33	8012	Red brown
Acc 95	160.67	113.67	47.33	35.00	70.67	63.00	1011	Brown beige
Acc 96	65.33	28.67	16.00	15.33	75.67	25.67	6022	Brown olive
Acc 97	77.67	33.67	16.67	16.33	80.33	30.33	8016	Mahogany brown
Acc 100	19.33	165.33	57.67	40.33	70.33	75.67	1004	Golden yellow
Acc 105	84.33	30.33	6.00	18.67	93.33	33.00	8012	Red brown
Acc 117	12.67	68.33	10.67	29.00	91.33	50.33	8003	Clay brown
Acc 121	29.00	20.33	16.00	19.67	46.67	11.33	9005	Jet black
Acc 131	62.00	24.33	10.33	16.33	84.33	24.33	8012	Red brown
Acc 150	102.33	55.67	14.00	26.67	85.33	40.33	8007	Fawn brown
Acc 151	111.00	58.00	23.33	22.00	78.33	43.67	8002	Signal brown
Acc 175	77.67	42.00	29.00	15.67	63.33	30.67	8007	Fawn brown
Acc 177	132.00	78.67	34.00	27.33	74.67	52.00	8001	Ochre brown
Acc 179	13.67	13.00	15.67	172.00	29.33	6.67	5011	Steel blue
Acc 184	84.00	41.33	25.67	132.00	68.67	33.00	8028	Terra brown
Acc 190	29.33	27.33	24.00	102.33	44.67	13.33	9005	Jet black
Acc 197	31.00	28.00	34.00	168.00	37.00	14.33	9011	Graphite black
Acc 199	69.33	33.67	15.67	19.67	79.00	27.33	6022	Brown olive
Acc 200	19.00	19.00	23.00	242.67	23.00	9.00	9005	Jet black
l. s. d	21.00	20.94	30.99	137.46	26.84	8.81	-	-
CV (%)	15.0	25.0	81.00	128.00	24.00	15.90	-	-
*p*-value	<0.001	<0.001	0.107	0.046	<0.001	<0.001	-	-

Acc: accession, l. s. d: least significance difference, CV: Coefficient of Variation.

**Table 3:** The germination percentage among twenty-four (24) genotypes subjected to simulated drought conditions using 5% PEG compared to a control treatment using distilled water.

	Germination %	
Genotypes	CT	SD
Acc 25	55	35
Acc 55	51	55
Acc 61	52	37
Acc 78	58	63
Acc 82	56	43
Acc 87	65	49
Acc 95	52	46
Acc 96	55	18
Acc 97	62	49
Acc 100	43	53
Acc 105	41	61
Acc 117	51	29
Acc 121	46	24
Acc 131	46	58
Acc 150	42	21
Acc 151	47	43
Acc 175	43	39
Acc 177	56	56
Acc 179	43	54
Acc 184	57	44
Acc 190	50	25
Acc 197	55	46
Acc 199	45	30
Acc 200	40	28
*p*-value	0.010	<0.001
s. e. d	7.95	4.478
l. s. d	15.79	8.788
cv%	29.8	10.2

GP: germination percentage, NSG: number of seeds germinated, CT: controlled treatment, SD: simulated drought, s.e.d: standard error of difference, l.s.d: least significant differences, CV: coefficient of variation.

**Table 4:** Drought stress tolerance indices of 24 Bambara groundnut genotypes evaluated under non-stressed and drought stress conditions. TOL; Tolerance, MPI; Mean Productivity Index, STI; Stress Tolerance Index, GMP; Geometric Mean Productivity, YI; Yield Index, YSI; Yield Stability Index, DRI; Drought Resistance Index, LSD; Least significance difference, CV; Coefficient of Variation, *p*-value; probability value Yp; yield under well-watered condition, Ys; yield under drought conditions, SSI; Stress Susceptibility Index.

Genotypes	Yp (g Seedling^−1^)	Ys (g Seedling^−1^)	SSI	TOL	MPI	STI	GMP	YI	YSI
Acc 25	0.359	0.082	1.084	0.277	0.220	0.310	0.172	0.922	0.229
Acc 55	0.240	0.134	0.622	0.106	0.187	0.337	0.179	1.502	0.558
Acc 61	0.306	0.117	0.867	0.189	0.212	0.377	0.189	1.317	0.383
Acc 78	0.312	0.112	0.902	0.200	0.212	0.368	0.187	1.258	0.359
Acc 82	0.461	0.107	1.080	0.354	0.284	0.518	0.222	1.200	0.232
Acc 87	0.356	0.089	1.053	0.267	0.223	0.335	0.178	1.004	0.251
Acc 95	0.299	0.101	0.932	0.198	0.200	0.317	0.174	1.132	0.337
Acc 96	0.368	0.019	1.332	0.348	0.194	0.075	0.084	0.218	0.053
Acc 97	0.233	0.109	0.747	0.124	0.171	0.267	0.159	1.227	0.469
Acc 100	0.228	0.104	0.760	0.123	0.166	0.250	0.154	1.174	0.459
Acc 105	0.248	0.105	0.808	0.143	0.177	0.275	0.162	1.185	0.425
Acc 117	0.347	0.060	1.162	0.287	0.203	0.219	0.144	0.675	0.173
Acc 121	0.324	0.033	1.262	0.291	0.178	0.113	0.103	0.371	0.102
Acc 131	0.343	0.107	0.966	0.236	0.225	0.388	0.192	1.206	0.312
Acc 150	0.279	0.042	1.196	0.237	0.160	0.122	0.108	0.467	0.149
Acc 151	0.399	0.105	1.034	0.294	0.252	0.442	0.205	1.184	0.264
Acc 175	0.246	0.084	0.925	0.162	0.165	0.217	0.144	0.944	0.342
Acc 177	0.288	0.086	0.984	0.202	0.187	0.261	0.158	0.969	0.300
Acc 179	0.365	0.105	1.001	0.260	0.235	0.402	0.196	1.179	0.288
Acc 184	0.219	0.160	0.377	0.059	0.189	0.368	0.187	1.798	0.732
Acc 190	0.296	0.042	1.207	0.254	0.169	0.130	0.111	0.471	0.141
Acc 197	0.307	0.106	0.918	0.200	0.207	0.343	0.181	1.196	0.347
Acc 199	0.322	0.077	1.071	0.245	0.199	0.260	0.157	0.862	0.238
Acc 200	0.258	0.048	1.144	0.210	0.153	0.130	0.111	0.540	0.186
LSD	0.185	0.0365	0.935	0.135	0.034	0.127	0.080	0.089	0.043
CV%	32.5	3.2	11.500	12.23	5.000	31.000	6.300	8.200	9.300
*p*-value	<0.001	<0.001	<0.001	<0.001	<0.001	0.069	<0.001	<0.001	<0.001

**Table 5:** Summary of factor loadings, eigenvalues, Kaiser-Meyer-Olkin measure of sampling adequacy, percent and cumulative variation for physiological parameters: tolerance, mean productivity index, stress tolerance index, geometric mean productivity, yield index, yield susceptibility index, root dry mass, root fresh mass, germination velocity index, and mean germination time, traits assessed among 24 Bambara groundnut genotypes under non-stressed and drought-stressed conditions.

	Controlled Treatment					Simulated Drought				
Traits	PC 1	PC 2	PC 3	PC 4	PC 5	PC 1	PC 2	PC 3	PC 4	PC 5
TOL	−0.074	0.613	−0.776	0.019	0.121	−0.351	−0.091	0.900	−0.186	−0.021
MPI	0.689	0.657	−0.237	−0.177	−0.038	0.538	−0.370	0.717	−0.184	−0.111
STI	0.845	0.345	0.251	−0.280	−0.131	0.873	−0.375	0.206	−0.131	−0.150
GMP	0.854	0.272	0.292	−0.264	−0.146	0.903	−0.336	0.136	−0.100	−0.147
YI	0.720	−0.072	0.641	−0.184	−0.170	0.887	−0.241	−0.336	0.036	−0.080
YSI	0.451	−0.363	0.780	−0.075	−0.144	0.677	−0.085	−0.661	0.134	−0.010
RDM	0.689	0.657	−0.237	−0.177	−0.038	−0.031	−0.035	0.629	0.153	0.350
RFM	0.312	0.723	−0.606	−0.081	0.057	0.538	−0.370	0.717	−0.184	−0.111
GVI	0.402	0.186	0.119	0.790	0.374	0.250	−0.096	0.219	0.383	0.738
MGT	0.519	0.206	0.130	0.668	0.320	0.822	−0.239	−0.115	0.238	0.298
Red	0.592	−0.650	−0.435	0.048	−0.020	0.491	0.833	0.167	0.025	0.003
Green	0.494	−0.703	−0.383	−0.142	0.204	0.419	0.839	0.037	−0.255	0.055
Blue	0.326	−0.437	−0.154	−0.403	0.624	0.309	0.473	−0.086	−0.675	0.322
Hue (°)	−0.144	0.363	0.502	−0.404	0.491	−0.036	−0.587	−0.285	−0.569	0.238
Saturation (%)	0.435	−0.283	−0.332	0.477	−0.586	0.338	0.459	0.312	0.700	−0.200
Lightness (%)	0.590	−0.651	−0.434	0.042	−0.003	0.489	0.833	0.163	0.009	0.012
RAL	−0.296	0.525	0.275	0.303	−0.262	−0.287	−0.596	0.035	0.406	0.060
Germination (%)	0.332	0.045	0.361	0.623	0.322	0.787	−0.223	−0.266	0.110	−0.003
Eigenvalue	5.105	4.189	3.396	2.385	1.540	5.830	3.943	3.220	1.887	1.038
Variability (%)	28.359	23.272	18.869	13.251	8.555	32.387	21.906	17.887	10.485	5.768
Cumulative (%)	28.359	51.630	70.500	83.751	92.306	32.387	54.293	72.180	82.664	88.433

## Data Availability

Data associated with the current study are presented in the manuscript.
